# Natural history of pulmonary atresia with intact ventricular septum (PAIVS) and critical pulmonary stenosis (CPS) and prediction of outcome

**DOI:** 10.1007/s00404-020-05929-0

**Published:** 2021-02-14

**Authors:** Aline Wolter, Natalia Markert, Jan Sebastian Wolter, Andrii Kurkevych, Jan Degenhardt, Jochen Ritgen, Rüdiger Stressig, Christian Enzensberger, Ivonne Bedei, Carina Vorisek, Johanna Schenk, Oliver Graupner, Markus Khalil, Josef Thul, Christian Jux, Roland Axt-Fliedner

**Affiliations:** 1 Division of Prenatal Medicine, Department of Obstetrics and Gynecology, Justus-Liebig-University Giessen and University Hospital Giessen & Marburg, Giessen, Germany; 2 Department of Cardiology, Kerckhoff Heart and Thorax Centre, Bad Nauheim, Germany; 3 Fetal Cardiology Unit, Ukrainian Children‘s Cardiac Centre, Kyiv, Ukraine; 4 Praenatal Plus, Köln, Germany; 5 Department of Obstetrics and Gynecology, Klinikum Rechts Der Isar, Technical University of Munich, Munich, Germany; 6 Department of Paediatric Cardiology, Children‘s Hospital, Justus-Liebig-University Giessen and University Hospital Giessen & Marburg, Giessen, Germany

**Keywords:** Fetal echocardiography, Pulmonary atresia with intact ventricular septum, Critical pulmonary stenosis, Biventricular outcome

## Abstract

**Objectives:**

To analyse prenatal parameters predicting biventricular (BV) outcome in pulmonary atresia with intact ventricular septum/critical pulmonary stenosis (PAIVS/CPS).

**Methods:**

We evaluated 82 foetuses from 01/08 to 10/18 in 3 centres in intervals 1 (< 24 weeks), 2 (24–30 weeks) and 3 (> 30 weeks).

**Results:**

61/82 (74.4%) were livebirths, 5 (8.2%) lost for follow-up, 3 (4.9%) had compassionate care leaving 53 (64.6% of the whole cohort and 86.9% of livebirths) with intention to treat. 9 died, 44/53 (83.0%) survived. 24/38 (63.2%) with information on postnatal outcome had BV outcome, 14 (36.8%) non-BV outcome (2 × 1.5 circulation). One with BV outcome had prenatal valvuloplasty. Best single parameter for BV outcome was tricuspid/mitral valve (TV/MV) ratio (AUC 0.93) in intervals 2 and 3 (AUC 0.92). Ventriculo-coronary-arterial communications (VCAC) were present in 11 (78.6%) in non-BV outcome group vs. 2 (8.3%) in BV outcome group (*p* < 0.001). Tricuspid insufficiency (TI)-Vmax > 2.5 m/s was present in BV outcome group in75.0% (18/24) vs. 14.3% (2/14) in non-BV outcome group. Including the most predictive markers (VCAC presence, TI- Vmax  < 2.5 m/s, TV/MV ratio < cutoff) to a score, non-BV outcome was correctly predicted when > 1 criterion was fulfilled in all cases. After recently published criteria for foetal intervention, only 4/9 (44.4%) and 5/14 (35.7%) in our interval 2 + 3 with predicted non-BV outcome would have been candidates for intervention. Two (1 × intrauterine intervention) in interval 2, two in interval 3 reached BV outcome and one 1.5 circulation without intervention.

**Conclusion:**

TV/MV ratio as simple parameter has high predictive value. After our score, non-BV outcome was correctly predicted in all cases. Criteria for foetal intervention must further be evaluated.

## Introduction

Pulmonary atresia with intact ventricular septum (PAIVS) is a rare, heterogenous cardiac anomaly with an incidence of 4–5/100,000 live births [1]. Outcomes have improved over time, but are still guarded with reported one- and 5-year survival rates of 70–75% and 63–67%, depending on the type of postnatal circulation [2, 3]

While in Germany prenatal detection rate for congenital heart defect (CHD) is low with < 20% for all CHD and about 42% for severe CHD [4], in UK two-thirds of cases with PAIVS/critical pulmonary stenosis (CPS) are detected prenatally [5]. The rate of termination of pregnancy (TOP) in PAIVS/CPS is up to 60%, especially when univentricular (UV) outcome is supposed [1]. Therefore, prediction of circulation outcome is an important aspect for parental counselling. Prenatal parameters have been investigated in smaller cohorts and there is ongoing debate on best predictors of postnatal circulation [5–13]. Foetal pulmonary valvuloplasty is performed in a few centres to obviate the consequences of postnatal non-biventricular (BV) circulation; however, treated cases are few, outcomes are limited and best selection criteria undetermined yet [14].

The aim of our study was to describe outcomes in prenatally diagnosed PAIVS/CPS, to analyse foetal echocardiographic parameters for predicting BV outcome and growth rate of these during gestation in the largest cohort to date.

## Materials and methods

The initial database found 94 foetuses with a prenatal diagnosis of PAIVS/CPS between 01/2008 and 10/20,182,017 in three centres, division of prenatal medicine Justus-Liebig-University Giessen Germany, Praenatal plus in Cologne Germany and Fetal Cardiology Unit in Ukrainian Children's Cardiac Center in Kiew Ukraine.

Informed parental consent to anonymised analysis of the data was given and the study was approved by the local research ethics committee of the faculty of medicine of Justus-Liebig university in Giessen (protocol number 219/16).

Nine patients with additional intracardiac abnormalities, e.g. Ebstein‘s anomaly or cases dominated by TV dysplasia were excluded as well as two cases with twin–twin transfusion syndrome (TTTS) and one with Williams–Beuren syndrome (WBS) as the mechanism of PS is usually different in those cases.

The final cohort comprised 82 foetuses. Foetal echocardiography was performed according to guidelines of ISUOG by a segmental approach and defined anatomical planes with colour pulsed-wave Doppler [15]. 5 MHz, 7.5 MHz or 9 MHz sector or curved array probes were used (Toshiba Aplio 500, Toshiba Aplio XG, Toshiba Medical, Neuss, Germany, Philips EPIQ 7, Philips iU22, Canon Aplio i900,GE Voluson E10).Fetal karyotyping was offered and included chromosome analysis and fluorescent in situ hybridization for microdeletion 22q11.2. Parental counselling by pediatric cardiologists was part of the prenatal work-up. Data were collected from medical files, ultrasound images and videos, whenever available. A complete foetal echocardiography was assessed. In contrast to PAIVS, CPS was diagnosed when a pinhole jet flow through the pulmonary valve (PV) was present. In all cases, a holosystolic reversal of flow within the arterial duct was diagnosed. Presence of ventriculo-coronary-arterial communications (VCAC), was documented.

**Fig. 1:**
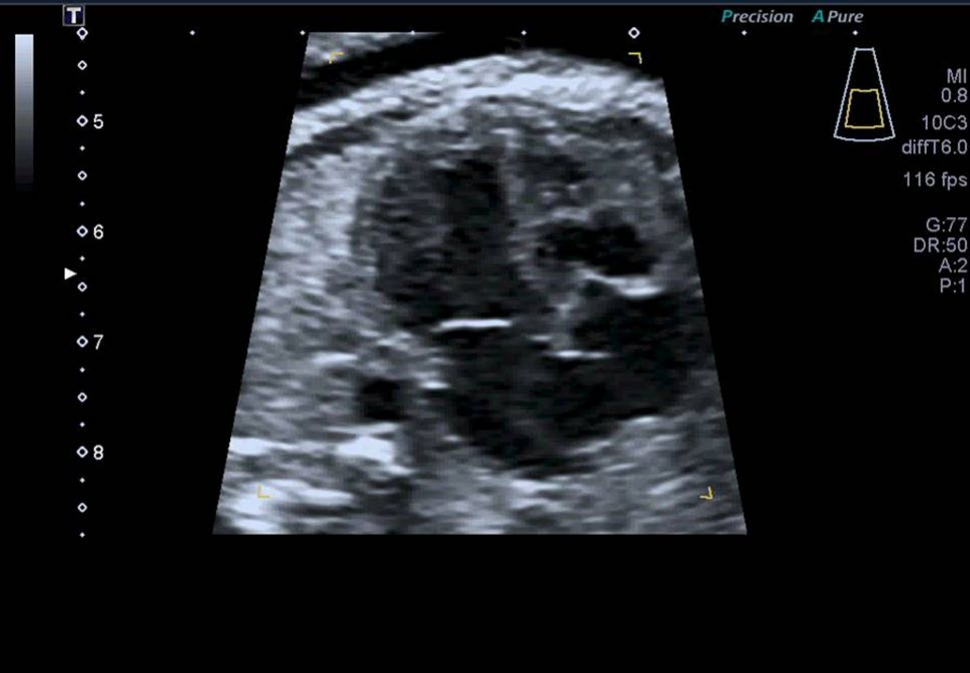
Example of fetus with PAIVS

An example of prenatal PAIVS is shown in Fig. 1.

**Fig. 2:**
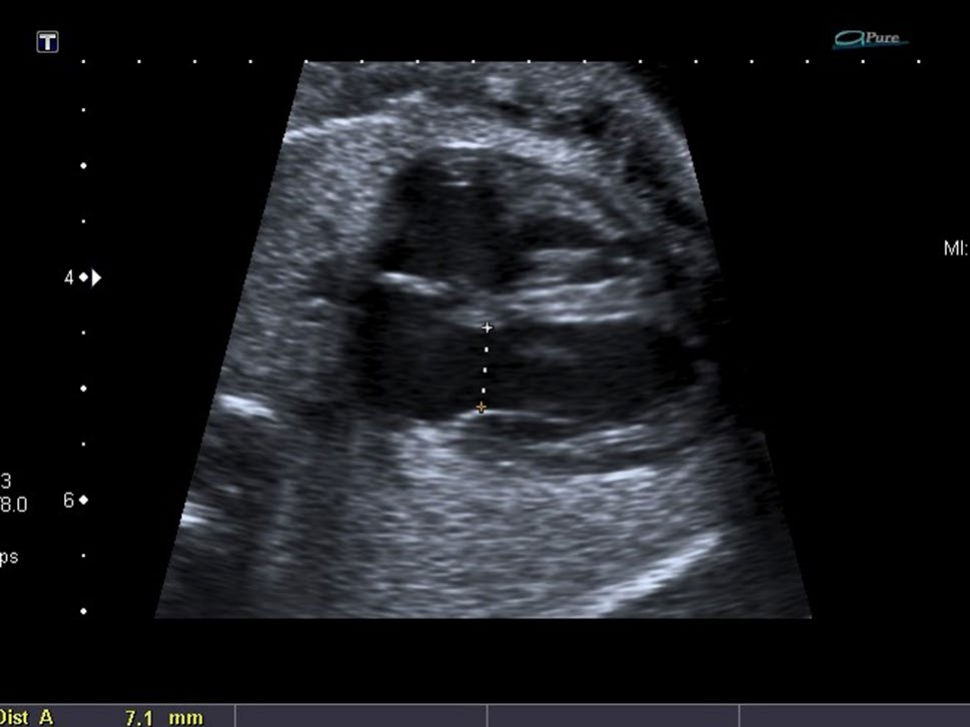
Example of measurement of MV

**Fig. 3:**
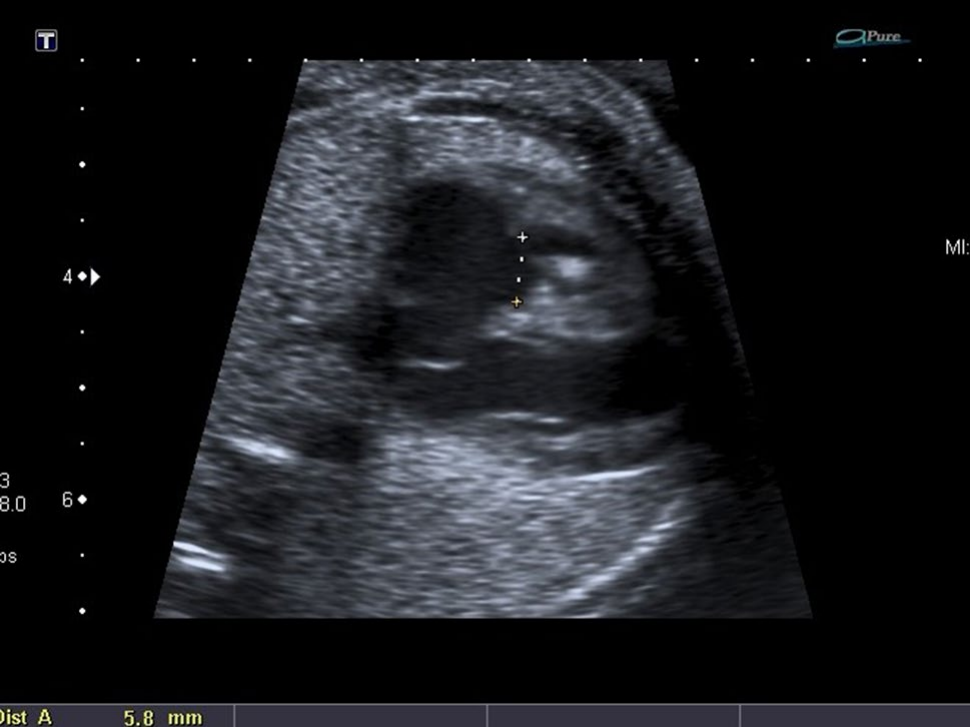
Example of measurement of TV

Tricuspid valve (TV) and mitral valve (MV) were measured at diastole when the diameter reached maximum before closing of atrioventricular valves, aortic and pulmonary valve at end-systole (Figs. 2, 3).

Most measurements were done prospectively. Missing parameters were retrieved from stored images/videos by repeated measurements and mean value was determined. Investigators were blinded to outcome. Right and left ventricular lengths were measured from the centre of atrioventricular valves to the endocardial surface in end-diastole. Z-scores were generated if required, using algorithm of Schneider et al. 2005 [16]. Continuous-wave Doppler of TI was recorded to measure peak velocity (Vmax). For data analysis, *R* version 3.5.2 was used.

Continuous variables are reported as mean ± SD or median (range) depending on the data distribution. Categorial data are expressed as frequencies and percentages (%) and compared among groups using Fisher’s exact test, *t*-test for normally distributed data or Mann–Whitney *U* test for not normally distributed data in interval-scaled variables. Normal distribution was tested using Shapiro test. Number of examinations and gestation week varied widely between the foetuses from different centres; therefore, data analysis was challenging. We used a model similar to that described by Gardiner and evaluated the measurements at three gestation intervals: interval 1 < 24 weeks, interval 2 from 24–30 weeks to and interval 3 > 30 weeks [5]. Mean for all z-scores and ratios was calculated for each foetus and interval. For echocardiographic parameters, Receiver-Operating-Characteristics (ROC) curves were calculated for establishing cutoff values. We included the most predictive parameters, TV/MV ratio, TI-Vmax < 2.5 m/s and presence of VCAC into a scoring system for non-BV outcome.

Longitudinal assessment of growth rate of pulmonary valve (PV), TV and RV lengths during gestation was calculated by measuring the change in dimension between the first and last fetal examination and dividing the result by number of weeks between both examinations [17].

## Results

### Description of prenatal cohort

Eighty-two pregnancies with prenatal diagnosis of PAIVS/CPS in three different centres were retrieved. There were seventy-nine (96.3%) singletons and three (3.7%) twins. Median maternal age was 29 (18–42) years. The rate for assisted reproduction was three of 82 (3.7%). Sixty-three foetuses presented with PAIVS (76.8%), nineteen (23.2%) had CPS.

Diagnosis of CHD was made in median 22.2 (12–31) weeks of gestation.

In one CPS, progression to PAIVS during pregnancy was observed. One patient had prenatal valvuloplasty in 25 + 5 weeks.

### Cardiac, extracardiac and genetic anomalies

Thirty-four (41.5%) foetuses had VCAC. Fifty-eight (71.7%) presented with TI. EFE within the right ventricle was present in twenty-five foetuses (30.5%) and restriction of the FO in six (7.3%) cases (Table 1). In five cases, VCAC was only diagnosed postnatally and three cases of VCAC were presumed prenatally, but not confirmed postnatally.

**Table 1:** Associated anomalies in our prenatal cohort with PAIVS and CPS

**PAIVS**	**63/82 (76.8%)**
CPS	19/82 (23.2%)
Chromosomal anomalies	1/82 (1.2%)
Extracardiac anomalies	3/82 (3.7%)
TI	58/82 (70.7%)
VCAC	34/82 (41.5%)
EFE	25/82 (30.5%)
Restrictive FO	6/82 (7.3%)
Prenatal intervention	1/82 (1.2%)

*PAIVS* pulmonary atresia with intact ventricular septum, *CPS* critical pulmonary stenosis, *TI*  tricuspid insufficiency, *VCAC *ventriculo-coronary-arterial communication, *EFE* endocardial fibroelastosis, *FO* foramen ovale

In seventeen (20.7%), foetuses karyotype was normal. We encountered one postnatally diagnosed trisomy 21.

In three (3.7%) patients, extracardiac anomalies were detected. One presented with ureteral stenosis and hypospadias; two had a single umbilical artery.

### Outcome

In eighteen of 82 (22.0%) cases, parents opted for TOP and two foetuses (2.4%) were lost for follow-up during prenatal period. One early IUD (1.2%) in 14 weeks occurred in PAIVS with small RV, absent A-wave in ductus venosus and increased nuchal translucency. Karyotype was normal; array diagnostic was refused. Sixty-one (74.4%) were livebirths, five (8.2%) were lost for follow-up postnatally, in three cases (4.9%) parents decided for compassionate care, leaving fifty-three (64.6% of the whole cohort and 86.9% of livebirths) with intention-to-treat. Nine (17.0%) of them died during follow-up; accordingly, intention-to-treat survival rate was forty-four of 53 (83.0%).

All demises within intention-to-treat group occured within nine months, seven of 9 (77.8%) died during the neonatal period. Two of them had non-BV outcome; in the other cases, circulation outcome was undetermined (Table 2). Median follow-up for surviving patients was 22.5 months.

**Table 2:** Details of deceased patients including three cases with compassionate care

	Cardiac diagnosis	Extracardiac anomalies	Delivery (weeks)	Preinter-vention-death	Operation	Reason for demise	Survival
1	PAIVS,VCAC, RVDCC	–	41 + 2	Yes	Parents decided for compassionate care after birth	Compassionate Care	2 days
2	PAIVS	–	35 + 0	Yes	–	Cardiac failure	1 day
3	PAIVS + VCAC, RAA	–	38 + 4	No	PDA stent not possible because of abnormal development of DA, BT Shunt, closing of VCAC because of hemodynamic worsening	Cardiac failure	4 months + 4 days
4	PAIVS, restrictive FO, VCAC	–	39 + 1	No	Rashkind, implantation 2 PDA stents, high-frequency perforation of PV, sequential balloon dilatation und implantation of RVOT-stent	Metabolic acidosis, post-intervention cardiac failure	13 days
5	PAIVS, VCAC	–	40 + 0	No	PDA stent, BT shunt, ECMO because of suspicion of shunt closure, reopening of shunt and stent implantation	Post-hypoxia and cerebral edema	47 days
6	PAIVS, tricuspid dysplasia, VCAC, restrictive FO	–	36 + 6	No	Unsuccessfull catheter intervention for opening PA, therefore emergent AP shunt, hybrid RVOT- opening und PA-RVOT-stent	Intraoperative electro-mechanical dissociation with exitus	4 days
7	PAIVS, VCAC	–	37 + 4	No	emergent BT shunt, PA closening because of VCAC, PA-reconstruction and central AP shunt	Cardiac failure	10 days
8	PAIVS,VCAC	–	38 + 5	Yes	Parents decided for compassionate care after birth	Compassionate care, hypoxia, cardiac failure	8 months + 25 days
9	PAIVS, VCAC	–	38 + 0	No	Palliative Rashkind to enlarge ASD for further medical care, no surgery or intervention with PV opening and RV decompression due to RVDCC	Cardiac failure, hypoxia	15 days
10	PAIVS, moderate TI	–	39 + 1	Yes	Parents decided for compassionate care	Compassionate care	1 day
11	PA:IVS, severe TI, severe hypoplasia PVs	–	39 + 1	Yes	Inoperable variant due to severe hypoplasia of pulmonary veins, medical treatment in cardiac ICU without intervention and catheterization	Cardiac failure	23 days
12	PAIVS, VCAC, RVDCC, IUGR, restrictive FO	–	37 + 1	NO	Rashkind, VCAC closure, right modified BT shunt	Cardiac failure	7 days

*PAIVS* pulmonary atresia with intact ventricular septum, *IUGR* intrauterine growth restriction, *RV* right ventricle, *DA* ductus arteriosus, *PV* pulmonary valve, *VCAC* ventriculo-coronary-arterial communication, *RAA* right aortic arch, *PDA stent* patent ductus arteriosus stent, *BT shunt* Blalock–Taussig shunt, *FO* foramen ovale, *RVOT* right ventricular outflow tract, *ECMO* extracorporeal membrane oxygenation, *AV* aortic valve, *RVDCC* right ventricle-dependent coronary circulation, *LV* left ventricle, *AP shunt* aortopulmonary shunt, *PV* pulmonary veins

In thirty-eight of 53 (71.7%) patients, information regarding BV/non-BV including 1.5 circulation was available.

Seven of them were patients with early neonatal death and in eight patients, outcome was still undetermined, mainly due to short follow-up at the time of evaluation.

**Fig. 4:**
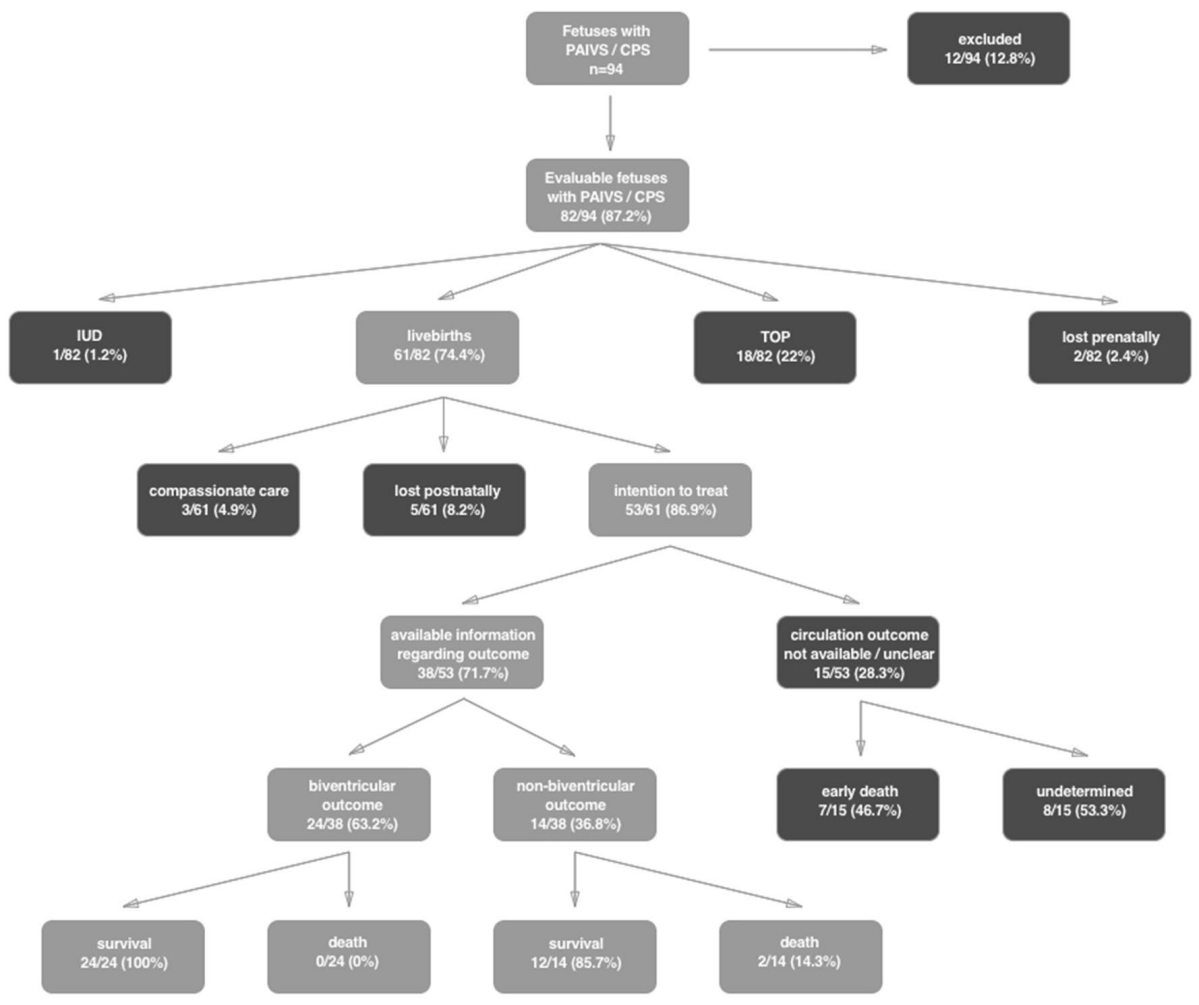
Overview of our cohort

Twenty-four of 38 (63.2%) patients had BV outcome, fourteen of 38 (36.8%) had non-BV outcome, two (1.4%) within this subgroup had 1.5 circulation. (Fig. 4).

Concerning perinatal data, delivery was at 38.43 (31.7–41.9) weeks. Nine (14.8%) were preterm deliveries including four within 32–34 weeks. A caesarean section was performed in 70%, most of them (66.7%) were primary caesarean sections. The main reasons for primary caesarean section were practical reasons as desire for planned delivery in case of remote home from the heart centre.

Median percentile of birth weight was 27.5. Perinatal data were comparable in both outcome groups.

Regarding intracardiac anomalies in BV versus non-BV outcome, there were significant more cases with VCAC within the non-BV group (11/14 (78.6%) versus 2/24 (8.3%) in BV outcome group) and more cases with TI-Vmax > 2.5 m/s in BV outcome group (18/24 (75.0%) versus 2/14 (14.3%) in non-BV outcome group). Two patients with VCAC had BV outcome, another patient with VCAC had 1.5 circulation (was documented in the non-BV outcome group). The rate for endocardial fibroelastosis (EFE) was not significantly different.

### Evaluation of parameters predicting BV versus non-BV outcome

We evaluated z-scores of PV, TV, pulmonary arteries, TV/MV ratio, PV/AV ratio, RV/LV length ratios and TI-Vmax in interval 1 (< 24 weeks), interval 2 (24–30 weeks) and interval 3 > 30 weeks.

In all intervals, ratios of TV/MV and PV/AV performed better in predicting circulatory outcome than zTV or zPV alone.

The best predicting parameter for BV versus non-BV outcome in interval 1 was PV/AV ratio (area under the curve (AUC 0.82) followed by TV/MV ratio (AUC 0.78)). Measurements in interval I were limited, because patients often were referred later.

In interval 2, the best predicting parameter was TV/MV ratio and RV/LV length ratio (both AUC 0.93). Cutoff for TV/MV ratio was 0.62 and Cutoff for RV/LV length ratio was 0.70 in this interval.

In interval 3, measurements were available in most patients. The best predicting parameter as well as in interval 2 was TV/MV ratio (AUC 0.92, cutoff 0.71), the second best was RV/LV length ratio (AUC 0.90, cutoff 0.68).

**Fig. 5:**
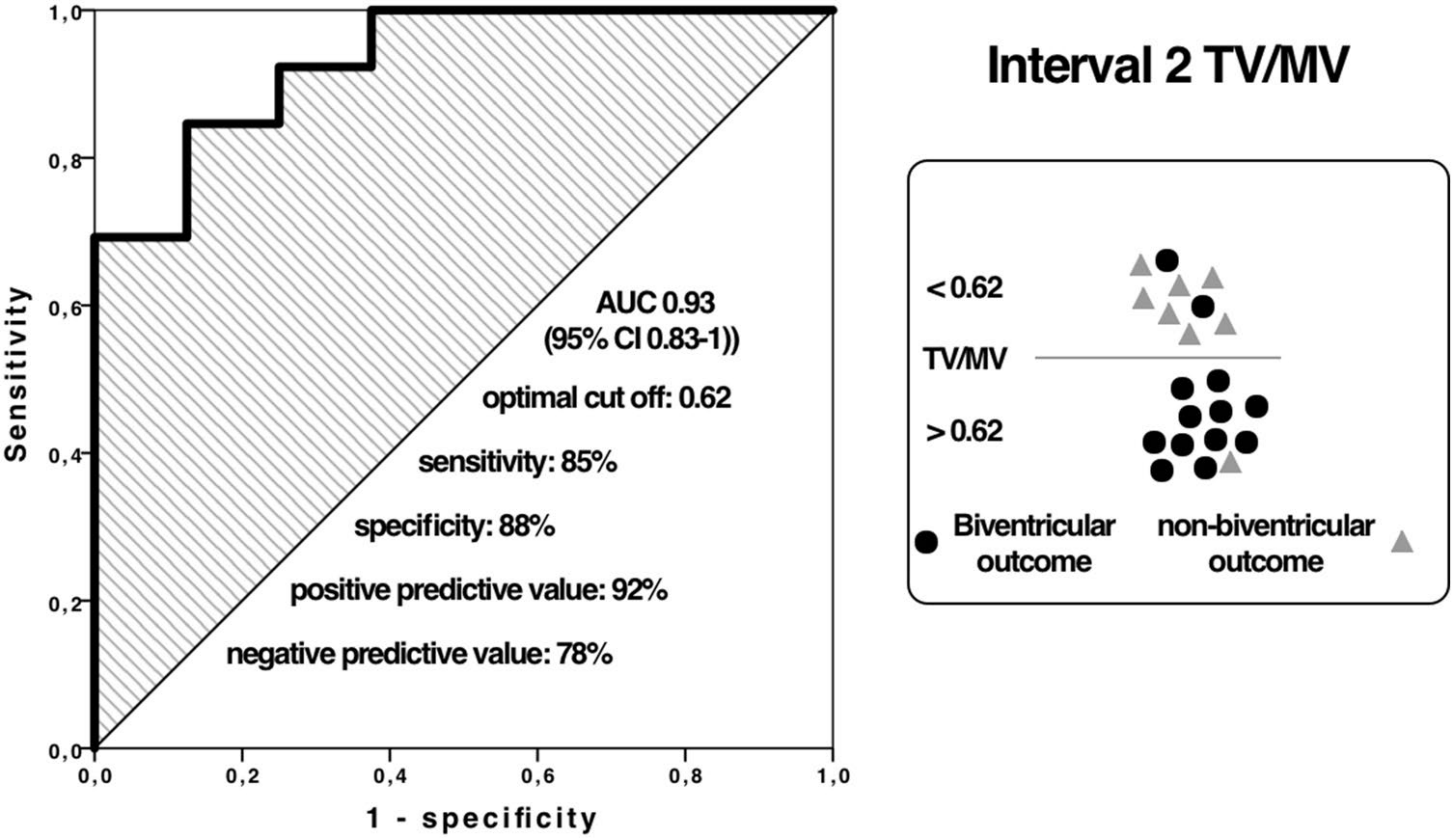
ROC curve for TV/MV ratio interval 2

**Fig. 6:**
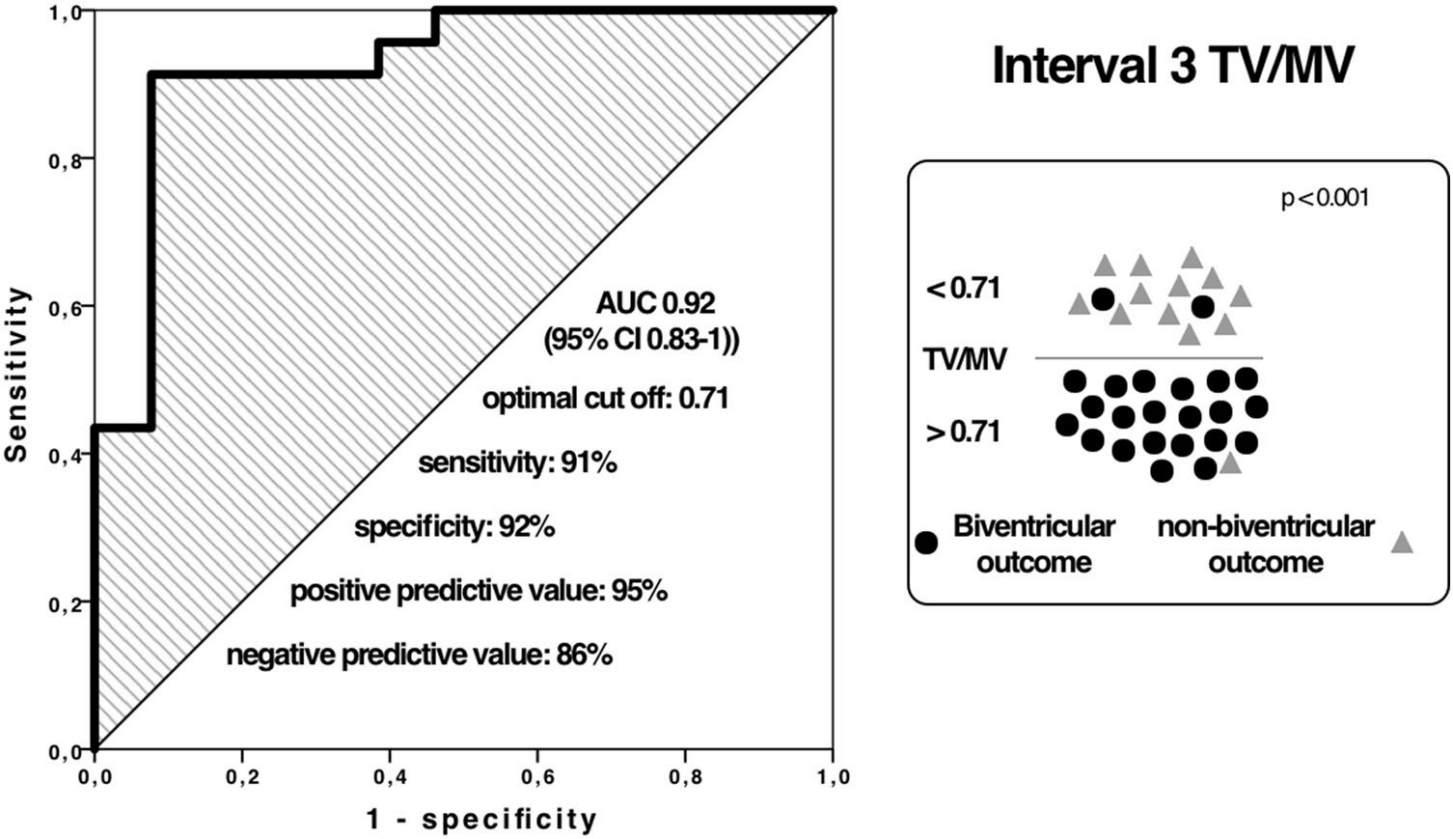
ROC curve for TV/MV ratio interval 3

Available measurement, AUC values, sensitivity, specificity, positive predictive value (PPV), negative predictive value (NPV) and cutoffs within the three intervals are summarised in Table 3. ROC curves for the best predictive parameter, TV/MV ratio, for intervals 2 and 3 with relevant number of measurements are shown in Figs. 5 and 6.

**Table 3:** Echocardiographic prediction parameters within 3 gestation intervals

Parameter		Interval 1	Interval 2	Interval 3
PV/AV ratio	*n*	16 (42.1%)	21 (55.3%)	35 (92.1%)
	AUC value	0.82	0.90	0.85
	sensitivity	88	85	74
	specificity	75	88	92
	PPV	78	92	94
	NPV	86	78	65
	Cutoff value	0.71	0.74	0.81
TV/MV ratio	*n*	15 (39.5%)	21 (55.3%)	36 (94.7%)
	AUC value	0.78	0.93	0.92
	sensitivity	75	85	91
	specificity	86	88	92
	PPV	86	92	95
	NPV	75	78	86
	Cutoff value	0.67	0.62	0.71
RV/LV length ratio	*n*	15 (39.5%)	18 (47.4%)	36 (94.7%)
	AUC value	0.57	0.93	0.90
	sensitivity	75	82	91
	specificity	71	100	77
	PPV	75	100	88
	NPV	71	78	83
	Cutoff value	0.67	0.70	0.68
Score > 1	*n*	11 (28.9%)	17 (44.7%)	29 (76.3%)
	AUC value	0.85	0.89	0.98
	sensitivity	1	1	1
	specificity	0.83	0.71	0.82
	PPV	0.81	0.83	0.90
	NPV	1	1	1

*PV* pulmonary valve, *AV* aortic valve, *AUC* area under the curve, *PPV* positive predictive value, *NPV *negative predictive value

One patient had prenatal pulmonary valve valvuloplasty in 25 + 5 weeks in our centre and BV outcome was achieved. The patient had absence of VCAC, TI-Vmax > 2.5 m/s. Mean zTV was − 4.42, TV/MV ratio was 0.53 and RV/LV length ratio was 0.60 in mean before intervention; so non-BV outcome would have been predicted after TV/MV ratio and RV/LV length ratio cutoff in our evaluation for this interval.

As described above, BV outcome was associated with TI-Vmax > 2.5 m/s and non-BV outcome with the presence of VCAC, so we combined these parameters with TV/MV ratio as best single parameter to a score. Non-BV outcome was correctly predicted in all cases if more than one criteria were fulfilled. False-positive rate for the prediction score was 17%.

**Fig. 7:**
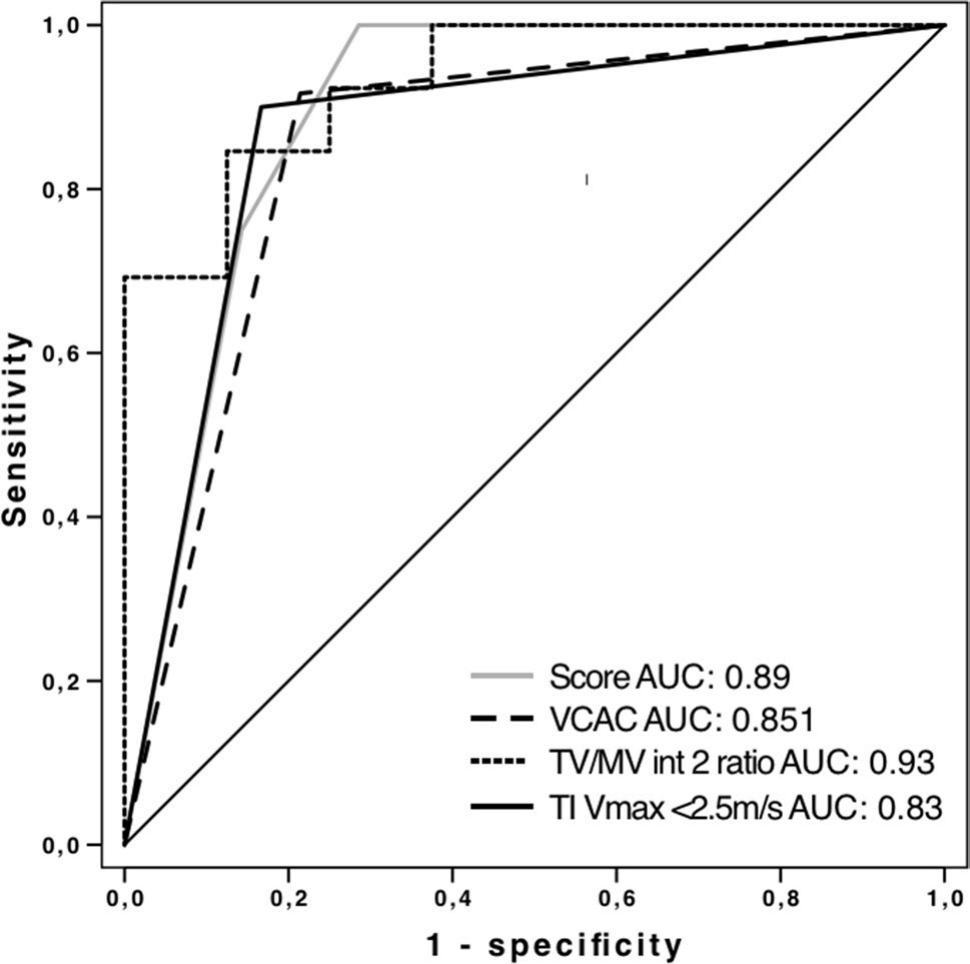
ROC curve for score parameters interval 2

AUC values, sensitivity, specificity, PPV, NPV for the score in the 3 different gestation intervals are summarised in Table 3. ROC curves of each predictive parameter, TV/MV ratio, TI-Vmax < 2.5 m/s and presence of VCAC for interval 2 with the biggest clinical impact are shown in Fig. 7.

### Longitudinal assessment of growth of PV, TV and RV lengths during gestation

There were no significantly different growth rates in patients with BV outcome and non-BV outcome for all three parameters. Only for TV, a tendency towards lower growth rates for patients with non-BV outcome (0.18 mm/week in non-BV outcome group versus 0.29 mm/week in BV outcome group, *p* value = 0.08) was seen.

## Discussion

The aim of the study was to describe survival rate and, second, to predict BV outcome by prenatal echocardiographic parameters.

Intention-to-treat survival rate (median follow-up 22.5 months) was 44/53 (83.0%) which fits to reported one-, 5- and 10-year survival rates ranging from 63 to 97% [2, 3, 18, 19]. Most deaths were neonatal deaths which is in line with others [18].

In our cohort, the best single, echocardiographic parameter to predict BV outcome was TV/MV ratio > 24 weeks. We included TV/MV ratio as best single parameter in combination with presence of VCAC and TI-Vmax < 2.5 m/s to a score. Non-BV outcome was correctly predicted in all our cases if more than one criteria were fulfilled. False-positive rate of the score is 17%, respectively, which is similar to previous data and should be considered for parental counselling [10].

In our cohort, almost two-thirds (63.2%) with evaluable circulation outcome had BV outcome, similar to published data [10, 20, 21]. TOP rate was high (22.0%). There exist reported TOP rates of up to 60%, and more parents will obviously opt for TOP when UV outcome is predicted. [1]

Data for prenatal predictors in PAIVS/CPS are limited with small cohorts of 15–34 analyzable cases [5–8, 10, 12, 20, 21] To our knowledge, this is one of  the largest cohorts in literature, with still small dataset of measurements, especially < 24 weeks.

Our results for TV/MV ratio showed AUC of 0.93 (cutoff 0.62) for 24–30 weeks and AUC of 0.92 (cutoff 0.71) > 30 weeks.

This is in line with Lowenthal et al. who find TV/MV ratio > 0.63 as strong predictor of favourable postnatal TV z-score in CPS/PAIVS [20]. Others also incorporated TV/MV ratio into their predictive scores [5, 10, 21]. Gardiner et al. developed scores for different gestation intervals including TV and PV z-scores and TV/MV ratio; they found a combination of TV/MV ratio with zTV as best predictor > 31 weeks [5]. Roman et al. developed a score by combining TV/MV ratio < 0.7, RV/LV length < 0.6, TV inflow duration > 31.5% and presence of sinusoids. When three criteria are fulfilled, non-BV outcome is predicted with sensitivity of 100%, specificity of 75%, PPV of 88% and NPV 100%. [10]. Recently, Gottschalk et al. reported a scoring system including (TR) < 2 m/s, right ventricle/left ventricle length ratio ≤ 0.6, and presence of VCAC with high sensitivity and specificity of 100% for prediction of UV circulation [12].

Gomez-Montes developed a score including TV/MV ratio ≤ 0.83, PV/AV ratio ≤ 0.75, tricuspid inflow duration/cardiac cycle length ≤ 36.5% and RV/LV length ratio ≤ 0.64. Three fulfilled criteria predict non-BV outcome with sensitivity of 100%, specificity of 92%, and both 100% if four criteria are fulfilled [21].

The only patient with prenatal pulmonary valvuloplasty in our cohort had TV/MV ratio of 0.53 and had BV outcome. After our cutoff of 0.62, non-BV outcome would have been predicted.

As already described by others in literature [6, 10, 12] we found significant more patients with VCAC within the non-BV outcome group. However, two patients with VCAC had BV outcome and one 1.5 circulation. Gardiner et al. also had one patient with VCAC and BV outcome and another with potential for BV outcome. According to Maeno et al., only 50% of VCAC are resulting in RV-depended circulation [9]. Successful right ventricular decompression depends on coronary arterial anatomy. Stenosis of a single coronary artery may not preclude successful right ventricular decompression; whereas, involvement of both arteries seems to be a contraindication. [22]

Prenatal detection of VCAC is challenging. Maeno et al. reported 7/13 (53.8%) correct prenatal diagnoses of VCAC. [9] In our cohort, prenatal diagnosis was failed in five cases of 34 (14.7%) and in three cases, VCAC were presumed prenatally, but not confirmed postnatally. TI-Vmax > 2.5 was associated with BV outcome and absence with UV outcome as described before [5, 7, 8, 20]. In our cohort, 75% with BV outcome had TI-Vmax > 2.5 m/s.

There exist few data concerning growth of RV structures during gestation.

While some showed significantly higher in-utero growth rate of TV in patients with BV outcome [6], we found also a tendency, but without significance.

In a recently published cohort of Tulzer et al. with 23 foetuses with intrauterine, pulmonary valvuloplasty at a median of 28 + 4 weeks, corresponding to our interval 2, the Roman score was retrospectively applied in 20/23 (87.0%) patients with prenatal valvuloplasty [23]. In ten of 20 (50%), UV outcome was predicted by applying this score. After pulmonary valvuloplasty, BV outcome was achieved in five of those 10 (50%) foetuses, in two circulation was yet undetermined, in two 1.5 circulation was reached and one with failed prenatal intervention had UV circulation [23].

However, in ten of 23 (43.5%) foetuses with prenatal valvuloplasty, the Roman score predicted BV outcome.

TV/MV ratio before intervention in the Tulzer cohort ranged from 0.62 to 0.97 (median 0.69). According to our TV/MV ratio, BV outcome would have also been predicted in these ten fetuses.

Knowing the fact that it is difficult to compare different cohorts, we applied the inclusion criteria of Tulzer for foetal intervention to our cases with predicted non-BV outcome after our score. Only four of 9 in interval 2 with predicted non-BV outcome would have been candidates for intervention. However, two of them reached BV outcome postnatally, one after intrauterine intervention and one without intervention. In interval 3, five of 14 would have been candidates for intervention. Two of them reached BV outcome and one 1.5 circulation without intervention, questioning the present selection criteria for foetal intervention. This issue was subject of controversy in the paper of the International cardiac intervention registry [24]. Suboptimal selection criteria for foetal aortic valvuloplasty have also been addressed by Gardiner et al. [25] It is difficult to apply published scores to different populations, because outcome also depends on postnatal decision-making and surgical performance which differs between centres. According to Tworetzky et al., foetal pulmonary valvuloplasty in PAIVS seems to improve right heart growth and postnatal outcomes for fetuses with moderate right heart hypoplasia, but there is an important learning curve [11]. It is an invasive procedure with risk of prematurity. Criteria for foetuses who might have a benefit must further be evaluated.

A limitation of our analysis is limitation of available measurements, small measurements with considerable error, combination of prospective and retrospective measurements and short-term postnatal outcome. Due to the retrospective data analysis, the lost-for-follow-up rate of patients is quite high and some echocardiographic variables included are without real blinding to outcomes (TI, presence of VCAC); so, there is potential for bias.

## Conclusion

To our knowledge, we evaluated one of the largest prenatal cohorts with PAIVS/CPS. TV/MV ratio is a simple single parameter with high predictive value. Prenatal diagnosis of VCAC is feasible in centres with expertise and is associated with non-BV outcome as already described before. Including the most predictive parameters, TV/MV ratio, TI-Vmax < 2.5 m/s and presence of VCAC into a scoring system, non-BV outcome was correctly predicted in all our cases if more than one criteria were fulfilled. Criteria for foetal intervention should be evaluated further in a prospective manner.
